# The Comparative Clinical Course of Pregnant and Non-Pregnant Women Hospitalised with Influenza A(H1N1)pdm09 Infection

**DOI:** 10.1371/journal.pone.0041638

**Published:** 2012-08-03

**Authors:** Gayle P. Dolan, Puja R. Myles, Stephen J. Brett, Joanne E. Enstone, Robert C. Read, Peter J. M. Openshaw, Malcolm G. Semple, Wei Shen Lim, Bruce L. Taylor, James McMenamin, Karl G. Nicholson, Barbara Bannister, Jonathan S. Nguyen-Van-Tam, the Influenza Clinical Information Network (FLU-CIN)

**Affiliations:** 1 Health Protection Research Group, Division of Epidemiology and Public Health, University of Nottingham, Clinical Sciences Building, Nottingham City Hospital, Nottingham, United Kingdom; 2 Centre for Perioperative Medicine and Critical Care Research, Imperial College Healthcare National Health Service Trust, London, United Kingdom; 3 Department of Infection & Immunity, University of Sheffield, Royal Hallamshire Hospital, Sheffield, United Kingdom; 4 Centre for Respiratory Infections, National Heart and Lung Institute, Imperial College, London, United Kingdom; 5 Department of Women’s and Children’s Health, Institute of Translational Medicine, University of Liverpool, Liverpool, United Kingdom; 6 Department of Respiratory Medicine, Nottingham University Hospitals National Health Service Trust, Nottingham, United Kingdom; 7 Department of Critical Care, Portsmouth Hospitals National Health Service Trust, Portsmouth, United Kingdom; 8 Health Protection Scotland, National Health Service National Services, Glasgow, United Kingdom; 9 Infectious Diseases Unit, University Hospitals of Leicester National Health Service Trust, Leicester Royal Infirmary, Leicester, United Kingdom; 10 Department of Health, Skipton House, London, United Kingdom; University of Hong Kong, Hong Kong

## Abstract

**Introduction:**

The Influenza Clinical Information Network (FLU-CIN) was established to gather detailed clinical and epidemiological information about patients with laboratory confirmed A(H1N1)pdm09 infection in UK hospitals. This report focuses on the clinical course and outcomes of infection in pregnancy.

**Methods:**

A standardised data extraction form was used to obtain detailed clinical information from hospital case notes and electronic records, for patients with PCR-confirmed A(H1N1)pdm09 infection admitted to 13 sentinel hospitals in five clinical 'hubs' and a further 62 non-sentinel hospitals, between 11th May 2009 and 31st January 2010.Outcomes were compared for pregnant and non-pregnant women aged 15–44 years, using univariate and multivariable techniques.

**Results:**

Of the 395 women aged 15–44 years, 82 (21%) were pregnant; 73 (89%) in the second or third trimester. Pregnant women were significantly less likely to exhibit severe respiratory distress at initial assessment (OR = 0.49 (95% CI: 0.30–0.82)), require supplemental oxygen on admission (OR = 0.40 (95% CI: 0.20–0.80)), or have underlying co-morbidities (p-trend <0.001). However, they were equally likely to be admitted to high dependency (Level 2) or intensive care (Level 3) and/or to die, after adjustment for potential confounders (adj. OR = 0.93 (95% CI: 0.46–1.92). Of 11 pregnant women needing Level 2/3 care, 10 required mechanical ventilation and three died.

**Conclusions:**

Since the expected prevalence of pregnancy in the source population was 6%, our data suggest that pregnancy greatly increased the likelihood of hospital admission with A(H1N1)pdm09. Pregnant women were less likely than non-pregnant women to have respiratory distress on admission, but severe outcomes were equally likely in both groups.

## Introduction

Increased mortality and morbidity in pregnant women have been observed during previous pandemics and influenza epidemics [1]–[4] and it is widely accepted that pregnancy is a risk factor for hospitalisation following influenza infection. [5], [6] The effects of maternal influenza infection are, however, not fully understood, though postulated to result from a combination of immunological and physiological changes. [7], [8].

In April 2009, an outbreak of novel influenza A(H1N1)pdm09 began in Mexico and on June 11^th^ the World Health Organization (WHO) declared a pandemic. The Department of Health, England, established an Influenza Clinical Information Network (FLU-CIN) at the outset in order to gather detailed clinical and epidemiological information from hospitalised A(H1N1)pdm09 cases in the UK. [9], [10] Previous reports from other, similar case series have demonstrated an increased risk of hospitalisation among pregnant women [11] but there are inconsistencies in the apparent severity of outcomes. Whilst some suggest that hospitalised pregnant women have a decreased risk of admission to intensive care [11]–[14], others report an increased risk of severe illness. [15], [16].

This study describes the characteristics of pregnant women in the FLU-CIN cohort hospitalised with confirmed A(H1N1)pdm09 infection. It compares pandemic influenza outcomes with non-pregnant women of the same age, adjusting for co-morbidities and illness severity at admission, which have not always been taken fully into account by other studies.

## Methods

The FLU-CIN project is described in further detail in an earlier paper. [9] FLU-CIN was an ‘emergency’ study with a purposive sampling frame based around 13 sentinel hospitals in five clinical ‘hubs’ (Nottingham, Leicester, Imperial College London, Sheffield and Liverpool), with contributions from a further 45 non-sentinel hospitals in England and 17 in Scotland, Wales and Northern Ireland. This included five children’s hospitals and five respiratory tertiary referral centres (three with facilities for Extra Corporeal Membrane Oxygenation (ECMO)). Children’s hospitals and tertiary referral centres were not mutually exclusive; one of three ECMO centres was a children’s hospital. Trained staff reviewed the hospital case notes and electronic records of patients with A(H1N1) pdm09 confirmed by real-time reverse-transcriptase polymerase chain reaction (PCR), and completed a common, standardised data extraction form. [9], [10] From this source cohort, all women aged 15–44 years were selected (childbearing age as defined by the National Association for Public Health Statistics and Information Systems). [17] Data collected included demographic characteristics, past medical history (including co-morbidities as denoted by the Charlson’s co-morbidity index [18], [19] and obesity) vaccination history, pre-admission care, clinical presentation, requirement for high dependency (Level 2) or intensive care (Level 3)admission, gestational age at admission, and maternal and foetal outcomes. High dependency (Level 2) care was defined as patients requiring more detailed observation or intervention (including support for a single failing organ system and those ‘stepping down’ from higher levels of care),and intensive (Level 3) care was defined as patients requiring advanced respiratory support alone or basic respiratory support together with support of at least two organ systems (including all complex patients requiring support for multi-organ failure). Any missing data were coded as dummy variables and reporting of information otherwise assumed to be complete.

Anonymised data were analysed using STATA (Version 11.0, StataCorp Inc.) and univariate analyses performed using logistic regression to compare factors associated with length of hospital stay, need for higher level care (admission to Level 2 or Level 3 care), death and combined severe outcomes (defined as Level 2/Level 3 admission or death) in pregnant and non-pregnant women of similar age. Multivariable regression analysis was conducted to adjust for factors that could potentially confound the relationship between pregnancy and pandemic influenza outcomes, using a conceptual model based on a combination of expert opinion and existing evidence. [10] Potential confounding variables selected for inclusion were co-morbidities (using Charlson’s Co-morbidity Index) [18], [19], recorded obesity (not classified under Charlson), in-hospital antiviral use and severity of illness at admission (indicated by severe respiratory distress and defined as severe breathlessness (e.g. unable to complete sentences in one breath, use of accessory muscles, supra-clavicular recession, tracheal tug or feeling of suffocation), and specified in the FLU-CIN questionnaire as CAT triage criteria A [20]). Missing values were coded separately as a dummy variable for inclusion in the multivariable logistic regression model. Sensitivity analyses were performed adding pre-admission antiviral use as a covariate in the model and restricting analysis to women without underlying co-morbidities.

## Results

### Characteristics of Pregnant Cohort

The FLU-CIN cohort consisted of 1,520 cases with PCR confirmed A(H1N1)pdm09 infection. Three hundred and ninety-five (26%) were women aged 15–44 years, 82 of whom (21%) were pregnant, compared with an expected prevalence of 6% based on the source population. [9], [10] One additional pregnant female identified in the full FLU-CIN cohort was excluded from the current analysis as she was aged 14 years.

On admission, six of the 82 pregnant women (7%) were in the first trimester, 33 (40%) in the second and 40 (49%) in the third (missing data  = 3). Fifty-nine (72%) pregnancies were reported to be single, and one multiple (missing data  = 22). Twenty-seven (33%) were admitted to hospital during the first wave of the pandemic and 55 (67%) during the second wave. The interval between onset of symptoms and admission ranged from 0–17 days (median 2.0; interquartile range 1–4; missing data  = 29). The main findings of the univariate analysis comparing the characteristics of pregnant and non-pregnant women aged 15–44 are presented in Table 1. Pregnant women were significantly less likely to exhibit severe respiratory distress at initial hospital assessment or require supplemental oxygen on admission than non-pregnant women of a similar age.

**Table 1:** Comparison of patient characteristics for pregnant and non-pregnant women of child-bearing age from the FLU-CIN cohort.

Characteristic	Value	Pregnant, n (%)	Non-pregnant, n (%)	OR* (95% CI)	P value
Age (years)	15	0 (0.0)	6 (1.9)	0.99† (0.96–1.02)	*P trend*0.370
	16–24	31 (37.8)	118 (37.7)		
	25–35	39 (47.6)	101 (32.3)		
	36–44	12 (14.6)	88 (28.1)	0.99† (0.96–1.02)	*P trend* 0.370 0.370
Charlson index score	0	62 (75.6)	152 (48.6)	1.00	
	1–2	19 (23.2)	151 (48.2)	**0.31 (0.18–0.54)**	
	3–5	1 (1.2)	10 (3.2)	0.25 (0.03–1.96)	
	>5	0 (0.0)	0 (0.0)	–	*P trend* **<0.001**
Obesity	–	3 (3.7)	15 (4.8)	0.75 (0.21–2.67)	0.662
Supplemental oxygen	–	11 (13.4)	87 (27.8)	**0.40 (0.20–0.80)**	**0.009**
Severe resp. distress$	–	27 (32.9)	156 (49.8)	**0.49 (0.30–0.82)**	**0.007**
CRP	<100	38 (46.3)	149 (47.6)	1.00	
	≥100	9 (11.0)	34 (10.9)	1.04 (0.46– 2.35)	0.929
	Missing	35 (42.7)	130 (41.5)	–	–
Pneumonia (radiological)	–	11 (13.4)	51 (16.3)	0.80 (0.39–1.60)	0.52
Admission within 2 days	–	16 (19.5)	82 (26.2)	0.74 (0.39–1.41)	0.36
	Missing	29 (35.4)	91 (29.1)	–	–
Pre-admission antiviral	–	8 (9.8)	46 (14.7)	0.63 (0.28–1.39)	0.25
In-hospital antiviral	–	61 (74.4)	253 (80.8)	0.69 (0.30–1.22)	0.20
Length of stay (days)	<2	15 (18.3)	47 (15.0)	1.00	–
	≥2	61 (74.4)	234 (74.8)	0.82 (0.43–1.56)	0.539
	Missing	6 (7.3)	32 (10.2)	–	–

* Unadjusted odds ratio.

$ Indicated by CAT triage criteria A^20^.

† Age fitted as a continuous variable in the logistic regression model.

### Underlying Co-morbidities

Pregnant women were significantly less likely to have other co-morbid illnesses than non-pregnant women. The most prevalent condition amongst those pregnant (approximately 80% of all recorded co-morbidities) was asthma, reported in 17 (21%) women. In contrast, asthma was reported by 128 (41%) non-pregnant women of similar age.

### Use of Anti-viral Medication

Fewer pregnant women were prescribed pre-admission or in-hospital anti-viral medication when compared with non-pregnant women, although neither of these findings was statistically significant. Sixty-one (74%) pregnant women were prescribed anti-viral medication in hospital, compared with 253 (81%) of non-pregnant women. There was insufficient information to comment on the time from symptom onset to treatment for either group. Nineteen pregnant women (23%) had seen a General Practitioner (GP) with influenza-like symptoms prior to admission, compared with 90 (29%) non-pregnant women (missing data = 196)(p = 0.29). Five of the nineteen (26%) pregnant women who had seen a GP were prescribed anti-viral medication in the community, compared with 24 of the 90 (27%) non-pregnant women (p = 0.98).

### Influenza Vaccination

Pandemic vaccination status was generally poorly recorded. Fifty-four pregnant women were admitted prior to the 23rd October 2009 and would either not have had the opportunity to be vaccinated or would not have seroconverted (even if vaccinated) prior to illness onset. Of the remaining 28, only two (7%) were reported to have received the vaccine, whereas seven (25%) were reported not to have received it (missing data = 19).

### Maternal Outcomes

Sixteen (20%) pregnant women were admitted within 2 days of symptom onset compared with 82 (26%) of non-pregnant women (p = 0.36, missing data  = 120). Eleven (13%) pregnant women had radiological evidence of pneumonia compared with 51 (16%) non-pregnant women (p = 0.53).

After adjustment for co-morbidities (including recorded obesity), severity of illness on admission, and in-hospital antiviral use, pregnant women were no more likely to require a hospital stay ≥2 days (OR 0.94, 95% CI 0.48–1.86, p = 0.865) or to experience severe outcomes including Level2/3 admission or death (OR 0.93, 95% CI 0.46–1.92, p = 0.808) (Table S1). In the sensitivity analysis where pre-admission antiviral use was added as an additional covariate, the results for all outcomes measures were almost identical (Table S2).There was also no statistically significant difference in the risk of severe outcomes (Level 2/3 admission or death) by wave of the pandemic when comparing pregnant and non-pregnant women (first wave unadj. OR 1.24, 95% CI 0.42–3.69, p = 0.69; second wave unadj. OR 0.78, 95% CI 0.32–1.88, p = 0.58).

Of the 11 pregnant women (13%) requiring Level 2/3 care, only three (27%) had underlying co-morbidities, two with diabetes mellitus and one with asthma. Ten (91%) required mechanical ventilation compared with 32 (70%) of the 46 non-pregnant women admitted to Level 2/3 care, however the difference was not statistically significant (p = 0.15). Seven of the 11 pregnant women had radiological evidence of pneumonia (64%), compared with 25 (54%) non-pregnant women in Level2/3 care (p = 0.58). Only one of the 11 (9%) pregnant women had been admitted to hospital within two days of onset (missing data  = 4) compared with 13 of 46 (28%) non-pregnant women (missing data = 17) (p = 0.14). Seven of the 11 (64%) pregnant women admitted to Level 2/3 care were prescribed anti-viral medication in-hospital compared with 38 of 46 (83%) non-pregnant women (p = 0.17); 2 (18%) pregnant women received treatment pre-admission, compared with 6 (13%) non-pregnant women (p = 0.66).

Three pregnant women died during admission, two with pneumonia and one following complications post delivery (coagulopathy, hypotension and cerebral infarction). There was no difference in the likelihood of death when compared with non-pregnant women (3 of 82 (4%) vs 11 of 313 (4%)) before (unadj. OR 1.04, 95% CI 0.28- 3.83) and after adjustment for co-morbidity, severity of illness on admission and in-hospital antiviral use (adj. OR 1.18, 95% CI 0.31- 4.53). In addition there was no difference in case-fatality between pregnant or non-pregnant women who were sick enough to require mechanical ventilatory support (2 of 10 (20%) vs. 7 of 32 (22%): unadj. OR 0.89, 95% CI 0.15–5.20).

Overall, pregnant women hospitalised with A(H1N1)pdm09 infection were no more likely to experience severe outcomes (Level 2/3 admission or death) when compared with non-pregnant women of a similar age (adj. OR 0.93, 95% CI: 0.46–1.92). Similarly, the sensitivity analysis considering only women without prior co-morbidities (Charlson index score = 0; pregnant = 62; non-pregnant = 152) and designed to exclude any residual confounding effects, also found no evidence that pregnant women had an increased risk of severe outcome either before (unadj. OR 0.92, 95% CI 0.47–1.83) or after adjusting for disease severity at admission (adj. OR 0.97, 95% CI: 0.39–2.45).

### Foetal Outcomes

Data regarding foetal outcomes were taken from free text notes. Sixteen babies were delivered during admission, ten to mothers admitted for predominantly obstetric reasons and four to mothers admitted for predominantly respiratory illness (missing data = 2), although clearly it is impossible to rule out influenza having a detrimental effect on a pregnancy and precipitating an obstetric issue. Three of the four mothers with apparent respiratory illness required Level 2/3 care and delivered pre-term babies, two requiring emergency caesarean section and one induced labour resulting in a still-birth. The fourth mother required emergency caesarean section but not Level 2/3 care, and delivered at term. In comparison, none of the ten mothers admitted predominantly for obstetric reasons required Level 2/3 care and only one delivered a pre-term baby by elective caesarean section.

## Discussion

This is the only study of which we are aware to present findings amongst pregnant women hospitalised during both waves of the 2009 pandemic in the UK. In addition, it is unique in its comparison of severe outcomes for pregnant and non-pregnant hospitalised cases of childbearing age with confirmed A(H1N1)pdm09, following careful adjustment for severity of illness at admission and other confounding variables.

The data collected were not independently verified and there was no attempt to obtain missing fields or follow-up cases, which may have resulted in some reporting bias. However, there was full extraction from case records, and comparison with non-pregnant women of the same age that also had A(H1N1)pdm09 infection, which illuminates the course of A(H1N1)pdm09 in pregnancy.

The high prevalence of pregnancy in this cohort of women aged 15–44 years, aligns with previous studies which suggest a marked increase in the likelihood of admission with A(H1N1)pdm09 when compared with non-pregnant women of similar age. [11], [15] In accordance with other published case series, [12], [13], [16], [21], [22] the majority of women were in the second and third trimesters of pregnancy, suggesting an increased risk of hospitalisation in the later stages of pregnancy, although this may be confounded by a failure to recognise some early pregnancies. Pregnant women were significantly less likely to have underlying co-morbidities than non-pregnant women of childbearing age, emphasising the importance of pregnancy itself as a risk factor for hospitalisation. But despite the increased likelihood of admission for pregnant women, we found little evidence of greater illness severity at initial hospital assessment, as suggested by our findings that pregnant women were less likely to present with respiratory distress or require supplemental oxygen on admission. This does not appear to be confounded by the use of antiviral medication, since pregnant women were, if anything less likely to receive this pre-admission. Other factors such as a lower threshold, or alternative primary indication for hospital admission (such as obstetric complication) may be of more importance. The reason for admission was, however, not always reported making it difficult to explore this further by stratified analysis. Three quarters (74%) of the pregnant cohort required a hospital stay of two days or more, suggesting that ‘front door’ anxiety for the pregnant patient may not fully explain the increased admission rate.

There was no evidence of a greater likelihood of severe outcomes in pregnant women as indicated by length of stay, requirement for Level 2/3 care or mortality. This held true even when adjusted for severity of illness on admission and underlying co-morbidities, or when comparing outcomes in women with no co-morbidities, through sensitivity analysis. The burden of mortality in other cohorts of pregnant women hospitalised with A(H1N1)pdm09 ranges from 1% [14] to 6% [13] and is not dissimilar from that observed in this case series. The risk of admission to intensive care does, however, appear to vary, some case series reporting this to be lower [11]–[14] and others higher [15] when compared with infected non-pregnant women of a similar age. This may be explained by different criteria for admission to Level 2/3 care which exist in different countries, although even when considering only the most seriously ill women who required mechanical ventilation, there was still no apparent increase in the risk of death for pregnant women in this cohort. Other studies have found pregnant women with confirmed A(H1N1)pdm09 in critical care experience higher rates of viral pneumonia despite a lack of difference in mortality [23], but no statistically significant difference was observed in this cohort. Although this suggests that once hospitalised, outcomes for women of childbearing age in the FLU-CIN cohort were similar regardless of pregnancy, caution must be exercised due to the smaller number of observations and potential lack of power with which to detect a true underlying effect.

Inconsistency in the estimated size of effect of pregnancy on severity of outcomes following A(H1N1) pdm09 infection may also reflect a marked variation in the prevalence of underlying co-morbidities and residual confounding; not all reports adjusted for presence of co-morbidities in the same way.[11]–[15] It is possible that differences in the ethnic composition of the populations considered may also have a role to play.

Despite the apparent lack of effect of pregnancy on severe outcomes once hospitalised, it seems that influenza infection in pregnancy nevertheless has serious implications for maternal health since ten of the 11 pregnant women admitted to Level 2/3 care in this cohort required mechanical ventilation, and only three had co-morbid illnesses. Further studies with greater power are however required in order to definitively conclude this. Where co-morbidities were reported for pregnant women, asthma was most prevalent, which again aligns with observations in other case series. [22], [24] Other data also suggest that A(H1N1)pdm09 cases with asthma experienced a reduced likelihood of severe outcome after admission. [9], [10].

Pregnant women were quickly identified as a priority group for both early anti-viral treatment and vaccination in the UK, as elsewhere. There was widespread availability of antiviral drugs in the community following establishment of a National Pandemic Flu Service in July 2009 [25], and whilst 74% of pregnant women in this cohort were prescribed anti-viral medication only 10% (8/82) received these in the community prior to admission. It is difficult to determine whether earlier treatment would have altered the observed outcomes, but delayed treatment has been associated with an increased risk of severe illness in pregnant women in previous hospitalised case series. [14], [16], [22] Although differences in antiviral drug uptake between pregnant and non-pregnant women are small, this suggests an ongoing need to emphasise the use of early anti-viral therapy on suspicion of influenza and promote its acceptability for use during pregnancy in a pandemic situation. [26] Evidence regarding the safety of Oseltamivir in pregnancy is, however, limited [27], [28] and further studies which focus on the risk of potential adverse events would clearly be of value.

Vaccination status was inconsistently recorded in medical notes and may in part be explained by the fact that the pandemic vaccine was not introduced in the UK until late October 2009, so 54 of the pregnant women in the cohort would either not have had the opportunity to be vaccinated, or would not have seroconverted (if vaccinated), prior to illness onset. Although there were large amounts of missing data, the fact that only 7% of those for whom the pandemic vaccine would have been available in time to have offered protection were noted to have had it, compared with 25% noted not to have had it, suggests that coverage was low, as observed in other hospitalised cohorts. [29], [30] This further highlights the need to emphasise existing public health policy, and for healthcare workers, especially GPs and midwives, to advocate for vaccination against influenza during pregnancy.

Most women did not deliver during admission and were therefore not followed up as part of the FLU-CIN protocol. Of the sixteen women who did deliver during admission, three of the four who were admitted primarily with respiratory illness delivered pre-term babies and two mothers died post-partum. In comparison there was only one pre-term delivery and one maternal death amongst the ten women admitted for predominantly obstetric reasons. Adverse foetal outcomes following maternal influenza infection have previously been described, [30], [31] and although these data must be interpreted with caution, they suggest a potential short-term impact of influenza infection on foetal as well as maternal health.

### Conclusions

As judged by the need for hospital admission, this study corroborates previous evidence that pregnancy itself was a risk factor for significant illness due to A(H1N1)pdm09 infection. There is, however, little evidence from our data to suggest that maternal outcomes were any more or less severe than for non-pregnant women of a similar age in the UK, even when severity of illness at admission (indicated by respiratory distress), additional underlying co-morbidities and in-hospital anti-viral use were taken into account. However, some caution must be exercised due to the small numbers in the study. Despite national recommendations for early anti-viral treatment and vaccination, the former were either not accessed or not offered to one quarter of the pregnant women in this cohort at any point in the care pathway; and vaccine uptake was low amongst those for whom it was available. Both have been shown to be effective in reducing the frequency [32] or severity of respiratory illness [4] in pregnant women and there is a need to continue to advocate and emphasise their role in optimal obstetric care.

## Supporting Information

Table S1 **Comparison of pandemic influenza outcomes for pregnant women and non-pregnant women of child-bearing age from the FLU-CIN cohort (multivariable regression analysis).**
(DOCX)

Table S2 **Comparison of pandemic influenza outcomes for pregnant women and non-pregnant women of child-bearing age from the FLU-CIN cohort (multivariable regression analysis adding pre-admission antiviral use as a covariate).**
(DOCX)
