# Supplementary material for: The Comparative Clinical Course of Pregnant and Non-Pregnant Women Hospitalised with Influenza A(H1N1)pdm09 Infection
Source: PLoS One. 2012 Aug 3;7(8):e41638. doi: 10.1371/journal.pone.0041638 (PMC3411676; doi:10.1371/journal.pone.0041638)
Supplement: Table S1 — Comparison of pandemic influenza outcomes for pregnant women and non-pregnant women of child-bearing age from the FLU-CIN cohort (multivariable regression analysis). (DOCX) [file pone.0041638.s001.docx]

## Table S1 - Comparison of pandemic influenza outcomes for pregnant women and non-pregnant women of child-bearing age from the FLU-CIN cohort (multivariable regression analysis)

| **Characteristic** | **Value** | **Pregnant , n (%)** | **Non-pregnant, n (%)** | **OR^$^ (95% CI)** | **P value** |
| --- | --- | --- | --- | --- | --- |
| Length of hosp stay | <2days | 15 (18.3) | 47 (15.0) | 1.00 |  |
|  | >2days | 61 (74.4) | 234 (74.8) | 0.94 (0.48-1.86) | 0.865 |
| Level 2/3 admission^^^ | - | 11 (13.4) | 46 (14.6) | 0.91 (0.43-1.93) | 0.816 |
| Death | - | 3 (3.7) | 11 (3.5) | 1.18 (0.31-4.53) | 0.853 |
| Severe outcome^*^ | - | 12 (14.6) | 49 (15.7) | 0.93 (0.46-1.92) | 0.808 |

^$^ Adjusted for co-morbidity (Charlson’s Co-morbidity Index^18,19^  and recorded obesity), severity of illness at admission (severe respiratory distress indicated by CAT triage criteria A^20^) and in-hospital antiviral use.

^ Level 2 (pregnant n=1, non-pregnant n=14), Level 3 (pregnant n=10, non-pregnant n=32)

^*^ Combined severe outcome (Level2/3 admission or death)
